# Behaviour of blood glucose diabetes type 2 on the cardiac stress test: a new paradigm? What is its importance?

**DOI:** 10.1186/1758-5996-7-S1-A242

**Published:** 2015-11-11

**Authors:** Jonathan Nícolas dos Santos Ribeiro, José Lucas Batista Calado Costa, Erik Pinheiro de Belém Carvalho, Isabella Taís Albuquerque Silva, Jessica Aimée Lins de França, Jacy Souto Maior Ferreira Neta, Maria de Fátima Monteiro, Cláudio Barnabé dos Santos Cavalcanti, Denise Maria Martins Vancea

**Affiliations:** 1 Escola Superior De Educação Física/Universidade De Pernambuco, Recife, Brazil

## Background

Cardiac Stress Test (CST) is a maximal exercise test inexpensive, wide applicability and effectiveness tests in analysis with diabetics. During a brief maximal exercise embodiment hepatic glucose production increases from two to five times increasing the glucose levels.

## Objective

To analyze the behavior of blood glucose of type 2 diabetics during exercise testing.

## Materials and methods

Pre experimental design study, duly approved by the Ethics Committee No. 775654. Through a sample test were selected 51 diabetic patients of both genders, aged between 50 and 70 yrs. who did not use insulin therapy and/or use of beta-blocker drugs. The CST was carried out under the supervision of a medical cardiologist, in the morning, with a maximum interval of two h between the last meal. A treadmill with incline option was used, obtaining the electrocardiographic recordings were used via 12-lead system, and protocols were selected individually. The capillary glycemic (CG) was measured before and immediately after every effort obtained in CST. Statistical analysis was performed using the Wilcoxon test and Spearman correlation, adopting a significance level of p=0.05.

## Results

CG rate above 150 mg/dL was 52.9%. The behavior of the CG immediately after every effort obtained in CST, showed a significant decline (175.2±83.2 vs 159.6±78.2 p=0.00). The heart rate (HR) Maximum evaluated showed significant correlations compared to the percentage of effort made (153.0±12.6 vs 97.1±6.2% rs=0.78 p=0.00), with the HR submaximal expected (153.0±12.6 vs 133.2±7.0 rs=0.55 p=0.00), and the maximal HR for (153.0±12.6 vs 157.3±8 3 rs=0.56 p=0.00).

## Conclusion

CG behavior presented itself contrary to the vast majority of existing literature. Apparently the action of exercise on CST and the route of independent glucose uptake of insulin are effective for descendants glycemic responses. However more studies are needed to investigate possible mechanisms responsible for this outcome. It also needs attention the realization of the extent of CG before and after CST, since the discrepancy (hyperglycemia/hypoglycemia) glucose levels by diabetics presented before the CST in order to avoid potential acute clinical complications related to diabetes.

**Figure 1:**
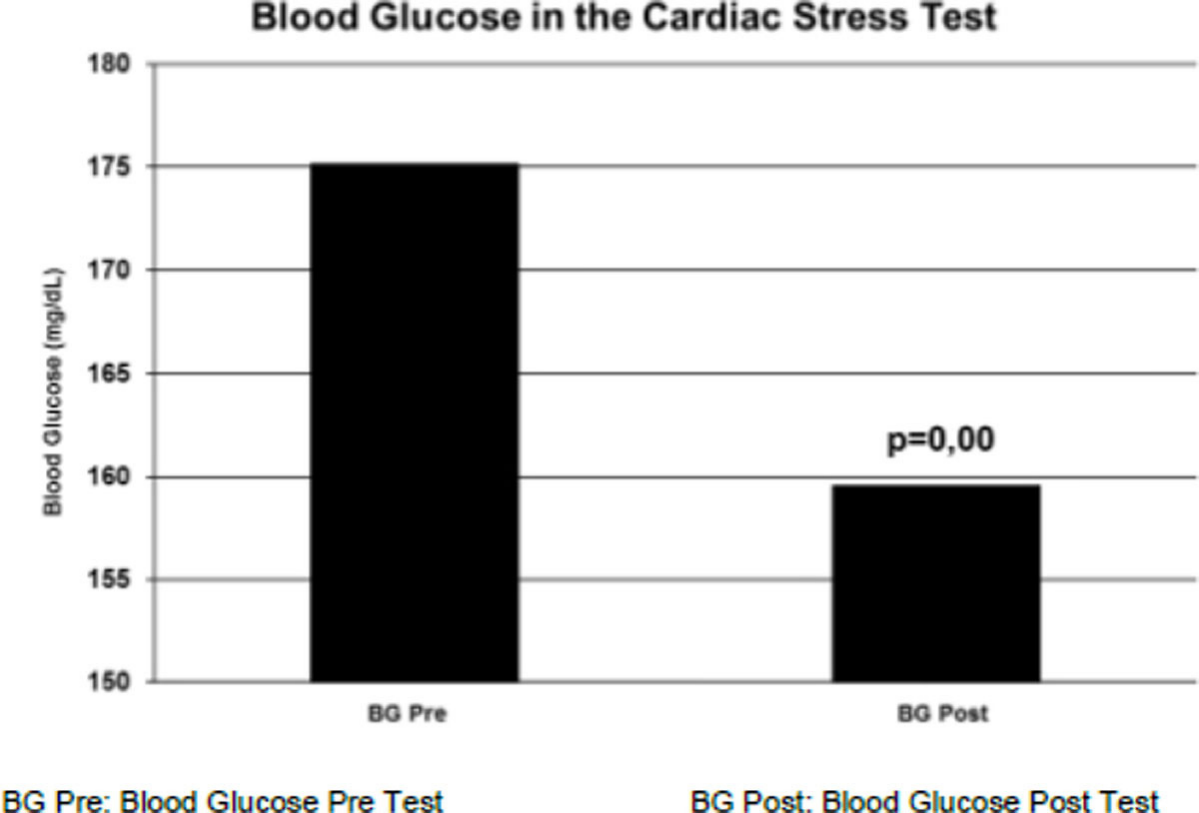
Behavior of the blood glucose in the cardiac stress test.

**Figure 2:**
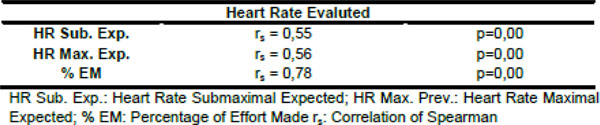
Correlation between heart rate evaluated and heart submaximal expected, heart rate maximal expected and percentage of effort made.

